# House dust mite drives biphasic immunomodulation of inflammatory dendritic epidermal cells via glucose metabolism in atopic dermatitis

**DOI:** 10.3389/fimmu.2026.1887390

**Published:** 2026-10-12

**Authors:** Ying Zhao, Cuie Gao, Xin Sui, Lan Ge, Huan Wang, Sisi Deng, Qiquan Chen, Lu Liu, Jing Tian, Tong Mu, Li Wang, Zhiqiang Song

**Affiliations:** 1 Department of Dermatology, The First Affiliated Hospital (Southwest Hospital) of Army Medical University, Chongqing, China; 2 Chongqing Municipal Health Commission Key Laboratory of Immunological Skin Diseases, Chongqing, China; 3 Institute of Immunology, College of Basic Medical Sciences, Army Medical University, Chongqing, China

**Keywords:** atopic dermatitis, glycolysis, IDECs, metabolism, oxidative phosphorylation

## Abstract

**Background:**

The impact of environmental antigens, specifically house dust mite (HDM) and *Staphylococcus aureus* enterotoxin B (SEB), on the metabolism of inflammatory dendritic epidermal cells (IDECs) and its role in atopic dermatitis (AD) pathogenesis is poorly understood. This study aimed to characterize IDECs metabolism in AD and study how antigen exposure drives metabolic reprogramming and immune responses.

**Methods:**

We first analyzed the single-cell RNA transcriptome sequencing data to explore metabolic profiles in IDECs from AD patients. Subsequently, we evaluated the effects of HDM alone or in combination with SEB for 24 h on the energy metabolism, maturation and cytokines secretion of monocytes-derived IDECs (moIDECs) *in vitro*. Finally, we investigated the dynamic changes of functional and glucose metabolism in moIDECs *in vitro* treated with HDM at different time points (6 h, 24 h, 48 h, 72 h).

**Results:**

In AD skin lesions, inflammation- and metabolism-related genes of IDECs are abnormally expressed, and multiple metabolic pathways, including glycolysis/gluconeogenesis, are significantly dysregulated. HDM alone or in combination with SEB activated the glycolysis, induced the upregulation of maturation markers (CD80/83/86) of moIDECs *in vitro*, and promoted the production of inflammatory cytokines (IL-1β, IL-6, IL-23, IL-10, IL-12 and TNF-α), whereas SEB alone showed no significant effect. Furthermore, we found that HDM dynamically regulated inflammatory immune response of moIDECs. In the early phase (6–24 h), a pro-inflammatory profile predominated, observed with peak levels of IL-6, IFN-γ, IL-18, IL-12, TNF-α and IL-23. This subsequently transitions into the late phase (48–72 h) characterized by an anti-inflammatory shift, marked by elevated IL-10, classical tolerogenic molecules (IDO1, PDL1, and ILT3), and a profile consistent with the potential to induce regulatory T cells (Tregs). Concurrently, metabolic adaptations were observed, with glycolysis peaking at 24 h.

**Conclusion:**

HDM-driven AD progression is correlated with changes in glucose metabolism and a biphasic immune shift in IDECs. Targeting HDM-responsive metabolic pathways like glycolysis might offer novel therapeutic strategies.

## 1 Introduction

Atopic dermatitis (AD) is a prevalent chronic inflammatory skin disease affecting up to 20% of children and 10% of adults globally, characterized by a complex interplay of immune dysregulation, epidermal barrier dysfunction, and genetic susceptibility (1). The AD epidermis contains a large number of epidermal dendritic cells (DCs). These cells use their dendrites and receptors to facilitate recognition, phagocytosis, and information transfer between skin surface antigens and the immune system (2). Unlike Langerhans cells or conventional DCs, inflammatory dendritic epidermal cells (IDECs) express high levels of the high-affinity IgE receptor (FcϵRI), which upon engagement triggers robust pro-inflammatory cytokine production and T-cell polarization (3). Moreover, IDECs have been directly implicated in the maintenance of chronic AD inflammation and correlate with disease severity. Therefore, understanding the function of IDECs is particularly relevant for deciphering the mechanisms that sustain AD pathology.

In AD skin lesions, a large number of monocyte-derived IDECs (moIDECs) are recruited to the epidermis from peripheral blood (2). They can release a large number of inflammatory factors and chemokines after activation, playing a central role in the initiation and maintenance of skin allergy (4, 5). Critically, the function of immune cells, including IDECs, is intrinsically linked to their metabolic state. Metabolic reprogramming—particularly a shift toward aerobic glycolysis—is recognized as a hallmark of activated, pro-inflammatory immune cells, providing the necessary bioenergetic and biosynthetic support for cytokine production and proliferation (6, 7). Therefore, IDECs are likely to adapt their metabolic profiles to generate sufficient energy for their highly activated inflammatory state.

The AD skin is perpetually exposed to a barrage of exogenous antigens that shape its immune pathology. The house dust mite (HDM) is a major aeroallergen whose proteolytic activity not only directly damages the epidermal barrier but also drives potent Th2-biased immune responses (8). Intriguingly, emerging evidence suggests that protease allergens like HDM can activate neuroimmune pathways, including sensory neurons, to promote sensitization, highlighting a complex, multi-cellular pathogenic cascade (9). *Staphylococcus aureus* colonizes over 70% of AD lesions and secretes a range of virulence factors, most notably staphylococcal enterotoxin B (SEB) (10). As a superantigen, SEB potently activates T cells by bridging the T-cell receptor and the co-stimulatory molecule CD28, leading to a massive release of inflammatory cytokines (11). These cytokines, in turn, can disrupt epithelial barrier integrity. These environmental challenges not only drive AD pathology but may also profoundly influence the metabolic state of key immune cells such as IDECs, though this connection remains poorly understood.

Our preliminary data show that the TLR2-mediated activation drives glycolytic metabolism in moIDECs (12).However, the specific immunometabolic effects of the natural antigen HDM, and the superantigen SEB, are entirely unknown. Furthermore, approximately half of AD patients spontaneously develop tolerance to HDM. Given that dynamic metabolic shifts are crucial for the transition of DCs from an immunogenic to a tolerogenic state, we postulate that HDM may not act as a simple, static activator. Instead, it might dynamically regulate the metabolic state of IDECs over time, potentially inducing a late-phase immunosuppressive phenotype that could contribute to the chronic history of AD.

This study aims to delineate the effects of HDM and SEB on moIDECs metabolism and function. We will investigate whether these antigens reprogram moIDECs metabolic pathways and, specifically, whether HDM can drive a functional shift in moIDECs from pro-inflammatory to anti-inflammatory through dynamic metabolic control. These insights may reveal novel immunometabolic mechanisms underlying AD pathogenesis.

## 2 Methods

### 2.1 Collection and analysis of single-cell RNA transcriptome sequencing data

We downloaded single-cell transcriptome sequencing data of 8 AD and 7 HC skin from the Caucasian population from the Gene Expression Omnibus database (GSE 153760) (13). The myeloid cells were annotated as DC-1, DC-2, DC-3, LC, and Mac. We have retained this original nomenclature for consistency with the source data. Seurat (4.0.1) was used for subsequent calculations of the downloaded count matrix in R (4.0.2). For analysis of scRNA-seq data, please refer to **Supplementary Methods**.

### 2.2 Induction and stimulation of moIDECs models

Human peripheral blood was collected from healthy volunteers (n = 15); the individuals recruited were from the Southwest Hospital of the Army Medical University. The study was approved by the Ethics Committee of Southwest Hospital (Approval number KY202258). Isolated peripheral blood mononuclear cells (PBMCs) from healthy individuals who acquired written informed consent. The *in vitro* cell model of moIDECs has been induced as described in previous studies (4, 14), and the induction steps of this model are as follows. PBMCs were obtained from buffy coats of healthy individuals by Ficoll density gradient centrifugation (800 g, 20 min). CD14^+^ monocytes were isolated magnetically by positive selection using a magnetic cell sorter (CD14 MicroBeads and MS+ columns (Miltenyi Biotec, Bergisch Gladbach, Germany), according to the manufacturer’s protocol. We seeded 1 × 10^6 cells in each well of a 24-well plate, resuspended in 1 mL IMDM (Gibco, Carlsbad, CA, USA) + 10% FCS (Gibco, Carlsbad, CA, USA), in the presence of 500 IU/mL human recombinant GM-CSF (Sanofi US, Bridgewater NJ), 75 ng/mL IL-4 (Miltenyi Biotec, Bergisch Gladbach, Germany), 10 μg/mL IgE (Abcam, Cambridge, MA, USA) and 10 μmol/L β-mercaptoethanol (Sigma-Aldrich, St. Louis, Mo). Differentiation of CD14^+^ monocytes into moIDECs over 7 days. The moIDECs were stimulated with 50 μg/mL purified HDM extract (Greer Labs, Lenoir, NC, USA) alone or in combination with 100 ng/mL SEB (MCE, Princeton, NJ, USA) 24 h. Using 50 μg/mL purified HDM extract to stimulate moIDECs for 6, 24, 48, and 72 h. For glycolysis inhibition experiments, moIDECs were pretreated with 10 mM 2-deoxy-D-glucose (Sigma-Aldrich, St. Louis, Mo) for 2 h prior to HDM stimulation.

### 2.3 Detection of surface activation markers of moIDECs

Samples were measured on Gallios flow cytometer (Beckman, Miami, FL, USA) and analyzed by FlowJo (Treestar, Inc., Ashland, OR, USA). Antibodies used were as follows: PE anti-human CD83, BrilliantViolet 421™ anti-human CD86, FITC anti-human CD80, PE/Cyanine7 anti-human CD1a, BrilliantViolet 510™ anti-human CD14, APC anti-human CD206, 7-AAD Viability Staining Solution (all purchased from BioLegend). *In vitro*, human moIDECs were identified as CD1a^+^ CD206^+^ CD14^-^ live cells.

### 2.4 Assessment of glucose and lactate

The level of glucose and lactate in cell supernatants collected after 24 h of stimulation were measured using a glucose detection kit (Sigma-Aldrich, St. Louis, Mo, USA) and a lactate detection kit (Sigma-Aldrich, St. Louis, Mo, USA), according to the manufacturer’s protocol.

### 2.5 Targeted energy metabolomics study

Metabolites were extracted from the samples. For metabolomic studies, an ultra-high performance liquid chromatography tandem mass spectrometry (UPLC-MS/MS) was used for separation and subsequent analysis. To effectively determine the universal variation trends in a class of bioactive metabolites, we set up the absolute value of VIP > 1, and P < 0.05 as screening thresholds to identify differential metabolites. For details and analysis of targeted energy metabolomics, please refer to **Supplementary Methods**.

### 2.6 Oxygen consumption rate and extracellular acidification rate

OCR and ECAR reflect OXPHOS and glycolysis respectively. The moIDECs with or without stimulus were seeded in 96-well XF-microplates (Seahorse Bioscience, North Billerica, MA, USA) at 1 × 10^5 cells per well. After incubation for 24h, the medium was removed and replaced with unbuffered “seahorse medium” (XF Assay Base Medium, containing 10 mM glucose) heated at pH 7.4. ECAR was measured before and after treatment with glucose (10 mM), oligomycin (1 μM), 2-DG (50 mM). OCR was measured before and after treatment with oligomycin (1.5 μM), FCCP (2 μM), and Rotenone/antimycin A (both 0.5 μM), using a Seahorse XF 96-analyzer (Seahorse Bioscience, North Billerica, MA, USA) according to the manufacturer’s protocols. The data were analyzed according to the protocol recommended by Wave software.

### 2.7 RT-PCR

Total RNA was isolated from moIDECs using TRIzol reagent (Takara, Ohtsu, Shiga, Japan) according to the instructions of the manufacturer. The complementary DNAs were synthesized using the PrimeScriptTM RT reagent Kit (Takara, Ohtsu, Shiga, Japan). Real-time PCR was performed with TB GreenTM Premix Ex TaqTM II (Takara, Ohtsu, Shiga, Japan) on a CFX Connect (Bio-Rad). The primers are listed in **Supplementary Table S1**. The relative gene expression levels were corrected by normalization based on the β-actin expression levels.

### 2.8 CD4^+^ T-cells proliferation

CD4^+^ T cells were isolated from peripheral blood using the CD4^+^ T Cell Isolation Kit (Miltenyi Biotec), and the isolated cells were labeled with 5 mM CFSE (BioLegend). For coculture experiments, moIDECs were cocultured with allogeneic CD4^+^ T cells (DC/CD4^+^ T-cell ratio of 1:4) for 6 days. Cells were harvested and CFSE dilution was assessed by flow cytometry to quantification of CD4^+^ T cells.

### 2.9 LEGENDplex™ multiplex assay

Cytokines secreted by moIDECs and cytokines in the co-culture supernatants of moIDECs and CD4^+^ T cells were detected by LEGENDplex™ multiplex assay kit (BioLegend) according to the manufacturer’s protocol. Data were acquired from a Gallios flow cytometer (Beckman, Miami, FL, USA) and analyzed using the LEGENDplex software suite provided by the manufacturer.

### 2.10 Statistical analysis

For normally distributed data, differences between two groups were analyzed using the t test, and differences between multiple groups were analyzed using ANOVA. For nonnormally distributed data, differences between two groups were analyzed using the Mann-Whitney U test, and differences between multiple groups were analyzed using the Kruskal-Wallis test with Dunn’s test for multiple comparison correction. Correlation analysis was performed using Spearman’s correlation coefficient. R language or GraphPad Prism was used for data visualization. P < 0.05 was considered statistically significant.

## 3 Results

### 3.1 Abnormal expression of inflammation- and metabolism-related genes of IDECs in the AD group

We first profiled the composition of myeloid cells in lesional skin from AD patients and healthy controls (HC) using single-cell RNA sequencing. Myeloid cells were identified into 5 different subpopulations: DC-1, DC-2, DC-3, LC, and Mac (**Figure 1A**). We compared the myeloid cell composition of the AD group with the HC group and found that the DC-1 subpopulation accounted for the largest proportion of myeloid cells (AD: 66.2%, HC: 61.5%, **Figure 1A**). IDEC identified through marker genes CD1A, CD1C, FCER1A, and MRC1 were mainly included in the DC-1 subpopulation and were significantly increased in the AD group (**Figures 1B–D**). In the current DC taxonomy, the DC-1 population identified here which contains the IDEC subset corresponds most closely to the cDC2/monocyte-derived DC lineage. Then we further analyzed the differentially expressed genes (DEG) of IDECs in the AD group and the HC group. The results suggested that IDECs in the AD group show the expression of maturation related gene (CD86), antigen presentation-related genes (CD1B), inflammation-related genes (S100A8, S100A10), and metabolism-related genes (LDHA, LIPA, GLUL) were increased (**Figure 1E**). KEGG functional enrichment analysis of DEG in IDECs indicated that significantly different biological processes include: oxidative phosphorylation, leukocyte transendothelial migration, glycolysis/gluconeogenesis, and biosynthesis of amino acids (**Figure 1F**). The activities of some metabolic related pathways, such as glucose energy metabolism, glycolysis and pentose phosphate metabolism in IDECs from the AD group were significantly higher, while the activities of OXPHOS and lipid metabolism lower compared to the HC group (**Figures 1G, H**). The above results suggests that the skin inflammatory microenvironment may have reprogrammed the metabolic pattern of IDECs, and the upregulated glycolysis and downregulated OXPHOS are easily reminiscent of the metabolic switch after activation of IDECs.

**Figure 1:**
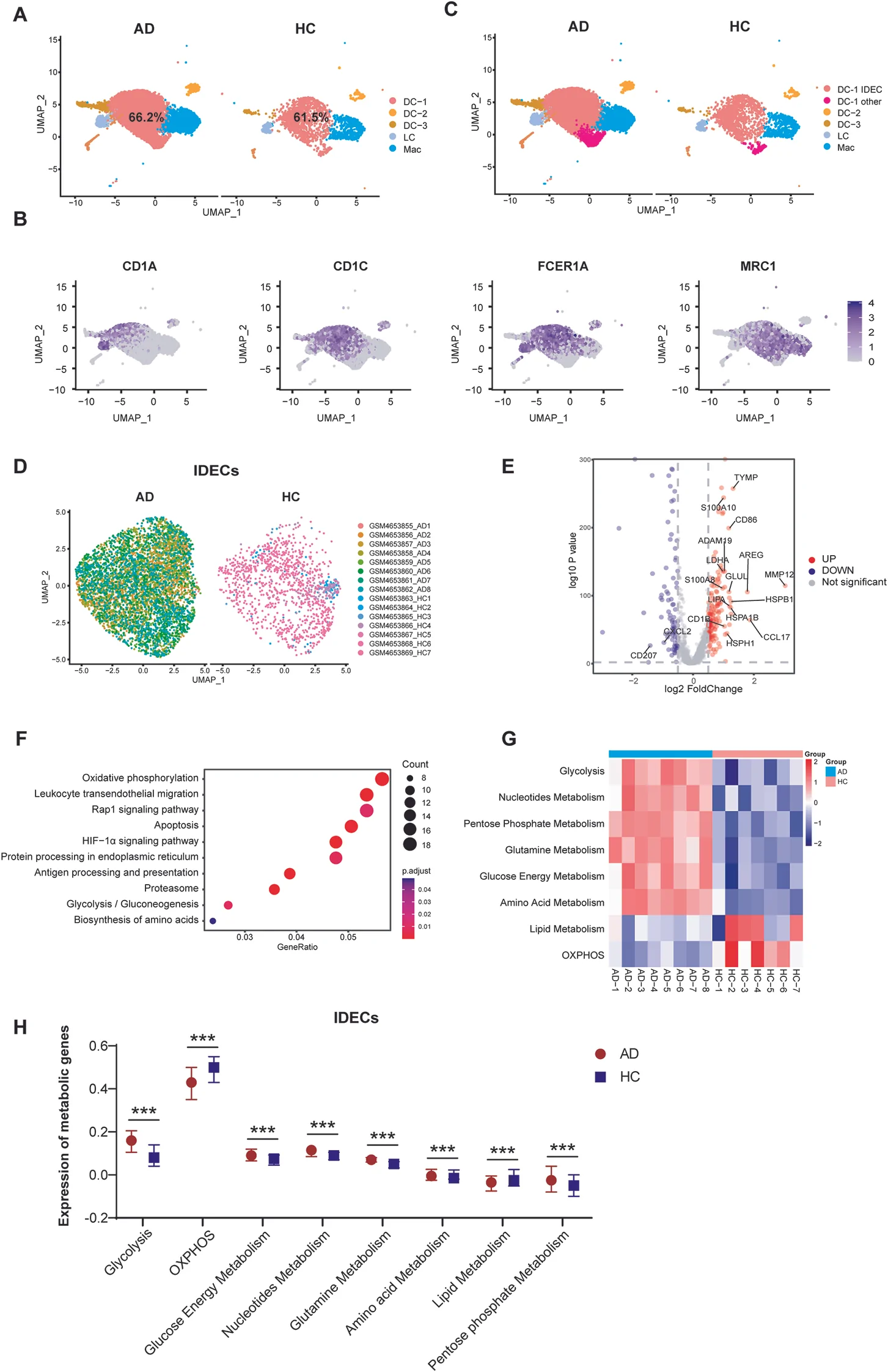
Abnormal expression of inflammation- and metabolism-related genes of IDECs in the AD group. **(A)** Similarity analysis of the transcriptome in myeloid cells from AD and HC groups using UMAP showed five different color-coded clusters. **(B, C)** Feature plots of expression distribution for marker genes of IDECs. Expression levels for each cell are color-coded and overlaid onto UMAP plots. IDECs were mostly included in the DC-1 subpopulation. **(D)** Comparison of UMAP plots of IDECs in the AD and HC groups. **(E)** Volcano plot showing genes differentially expressed in IDEC between AD and HC. log2(FC) values are shown on the x-axis and log10(p) values are shown on the y-axis. **(F)** KEGG analysis showing enriched biological processes of DEGs in IDECs between AD and HC. **(G)** Heat map of metabolic pathway activity in IDECs from AD and HC groups. **(H)** Comparison of the activities of glycolysis, OXPHOS, glucose energy metabolism, nucleotide metabolism, glutamine metabolism, amino acid metabolism, lipid metabolism, and pentose phosphate metabolism between the AD group and the HC group. Statistical analysis was performed using the Mann–Whitney rank sum test. ***P < 0.001.

### 3.2 HDM-stimulated moIDECs exhibited specific immunometabolic phenotypes

Having identified a dysregulated metabolic signature in moIDECs from AD lesions, we next sought to determine whether exposure to the major environmental antigens (HDM and SEB) in AD could directly drive such metabolic reprogramming. To this end, we established an *in vitro* model using monocyte-derived IDECs. Cells were stimulated with HDM, SEB, or their combination for 24 h, followed by an unbiased and systematic targeted energy metabolomics approach. A total of 66 energy metabolites were identified. An Orthogonal partial least squares discriminant analysis (OPLS-DA) model was used to determine the largest discriminant between the two groups. The negative control (NC) samples were well separated from HDM-stimulated samples and HDM+SEB-stimulated samples, but clustered together with SEB-stimulated samples (**Figures 2A–C**).

**Figure 2:**
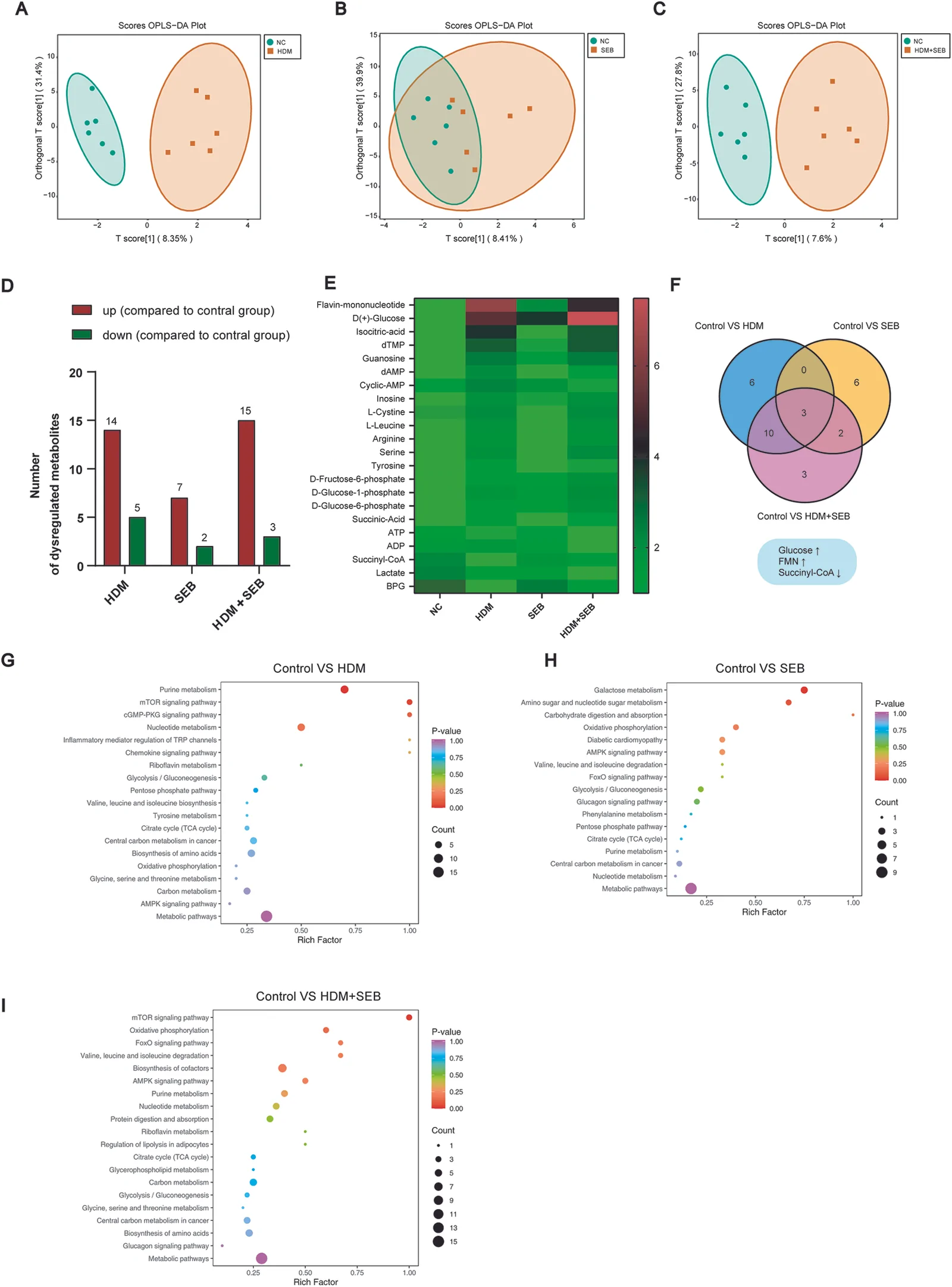
Targeted energy metabolomic results of moIDECs stimulated by HDM alone or combined with SEB. **(A)** Orthogonal partial least squares discriminant analysis (OPLS-DA) plot of metabolomics data, green circles are NC samples (n=6) and red squares are HDM samples (n=6). **(B)** Green circles are NC samples (n=6) and red squares are SEB samples (n=6). **(C)** Green circles are NC samples (n=6) and red squares are HDM+SEB samples (n=6). **(D)** Number of upregulated or downregulated metabolites at each treatment group. **(E)** Targeted metabolite profile of moIDECs in NC and different stimulation groups, intensity heat map plotted in nanograms of metabolites per sample (n = 6). Color keys represent fold changes in up-regulated (red) or down-regulated (green) differential metabolites compared to controls. **(F)** Venn plot of differential metabolites showing what is common and exclusive among the control and different stimulation groups. **(G–I)** KEGG pathway enrichment of differential metabolites in each treatment group compared to control.

The results revealed that in comparison to the NC group, the metabolites of the HDM group (14 upregulated and 5 downregulated), SEB group (7 upregulated and 2 downregulated), and HDM combined with SEB group (15 upregulated and 3 downregulated) changed significantly (**Figure 2D**). The glucose levels, flavin-mononucleotide (FMN) levels, amino acid levels (L-Cystine, L-Leucine, Arginine, Tyrosine, Serine) and nucleotide levels (dTMP, dAMP, Cyclic-AMP, Guanosine) of HDM-stimulated moIDECs were significantly increased, whereas SEB alone showed no significant effect (**Figure 2E**). The lactate levels of HDM-stimulated moIDECs were significantly decreased (**Figure 2E**). The differential metabolite patterns between the combined group and the HDM alone group were similar (**Figure 2E**). The common metabolites among the three groups included increased glucose and FMN, and decreased succinyl-coenzyme A (Succinyl-CoA) (**Figure 2F**). The KEGG pathway analysis showed that the common effect of different treatments on the metabolism of mo-moIDECs was primarily manifested in glycolysis/gluconeogenesis (**Figures 2G–I**). Taken together, our results indicated that HDM induces glycolysis/gluconeogenesis immunometabolic phenotype in moIDECs.

### 3.3 HDM shifted glucose metabolism by upregulating glycolytic genes and products in moIDECs

To investigate the changes in glucose metabolism of moIDECs after stimulation for 24 h, we examined the level of glucose and lactate in the extracellular supernatants of each group. We found that HDM significantly increased the lactate level in the extracellular supernatant, but did not affect the glucose level (**Figures 3A, B**). In addition, HDM-stimulated moIDECs showed higher expression of the key glycolysis enzymes related-genes (HK2, PFKM, LDHA) and lower expression of the key gluconeogenesis enzymes related-gene (FBP1) than the NC group (**Figures 3C–F**). At the same time, we found that the expression of glucose transporter 1 (GLUT1) and monocarboxylate transporter 4 (MCT4) increased in the HDM group (**Figures 3G, H**). Then, we assessed extracellular acidification rate (ECAR) and oxygen consumption rate (OCR) of moIDECs. Compared with the NC group, HDM alone or in combination groups significantly increased the glycolysis rate and glycolysis capacity in moIDECs, and inhibiting mitochondrial basal respiration and maximum respiration, whereas SEB alone showed no significant effect (**Figures 3I, J**). In addition, we found that HDM combined with SEB did not further change ECAR and OCR, compared with HDM group (**Figures 3I, J**). Taken together, these results implied that HDM-stimulated moIDECs undergo glycolytic metabolic reprogramming.

**Figure 3:**
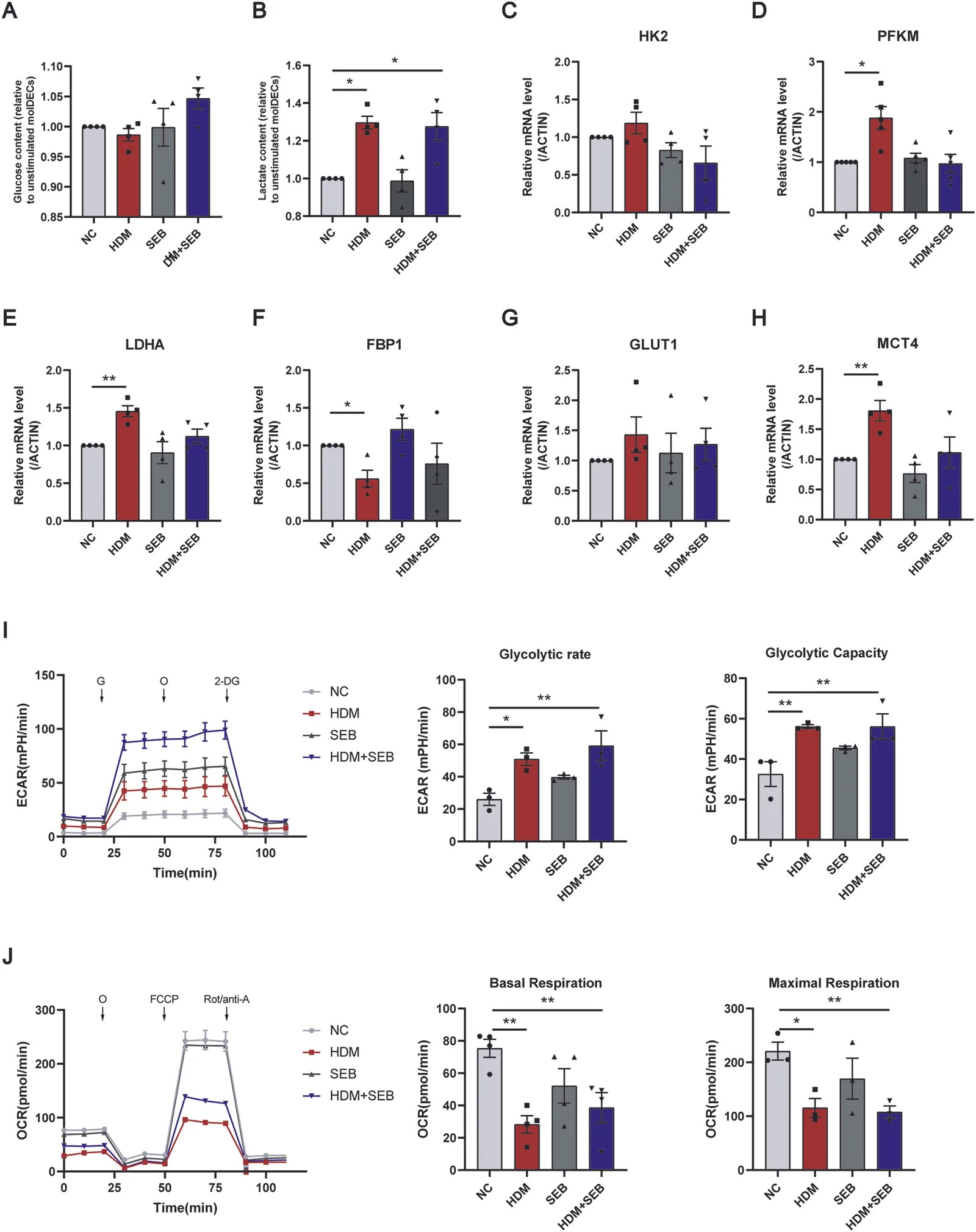
HDM shifted glucose metabolism by upregulating glycolytic genes and products in moIDECs. **(A, B)** The content of glucose and lactate in cell-free supernatant was quantified using glucose assay kit and lactate assay kit. Differential mRNA expression of HK2 **(C)**, PFKM **(D)**, LDHA **(E)**, FBP1 **(F)**, GLUT1 **(G),** MCT4 **(H)** in moIDECs from NC and different stimulation groups was analyzed by RT-PCR and normalized to housekeeping gene β-actin expression. **(I)** Glycolytic metabolic fluxes of injected glucose (G, 10 mM), oligomycin (O, 1 μM), and 2-DG (50 mM), including overall rate curves for extracellular acidification rate (ECAR), glycolytic rate, and glycolysis ability. **(J)** Metabolic fluxes of injected oligomycin (1.5 μM), FCCP (2 μM), and Rotenone/antimycin A (both 0.5 μM), including overall rate curves of oxygen consumption rate (OCR), basal respiration, and maximal respiration. No stimulation as negative control (NC). Bar graphs show mean ± SEM for at least 3 experiments. The significance was calculated using the one-way ANOVA. *P < 0.05; **P < 0.01.

### 3.4 HDM up-regulated maturation markers and enhanced cytokine secretion

Given the established link between cellular metabolism and effector function, and our observation that HDM selectively enhances glycolytic flux in moIDECs, we next assessed whether this metabolic reprogramming translates into specific functional changes. We stimulated moIDECs with HDM, SEB, or their combination for 24 h, and evaluated key indices of maturation and inflammatory response. TLR2 agonist Pam3CSK4 served as a positive control. Compared with the NC group, HDM alone or in combination groups significantly up-regulated the expression of moIDECs maturation marker molecules (CD80, P < 0.01; CD83, P < 0.001; CD86, P < 0.01) (**Figures 4A–C**). We also used 2-deoxy-D-glucose (2-DG) to inhibit glycolysis, and assessed the expression of CD83 as a maturation marker. In HDM-stimulated moIDECs, the upregulation of CD83 was markedly attenuated by 2-DG treatment (**Supplementary Figure S1**). At the same time, we found that concentrations of IL-1β, IL-6, IL-23, TNF-α, IL-10 and IL-12p70 were significantly increased after HDM alone or in combination groups, whereas SEB alone showed no significant effect (**Figure 4D**). Besides, HDM combined with SEB could not further upregulate the expression of mature marker molecules and the secretion of inflammatory factors, compared with HDM group (**Figures 4A–D**). These results showed that HDM promoted the maturation and inflammatory response function of moIDECs.

**Figure 4:**
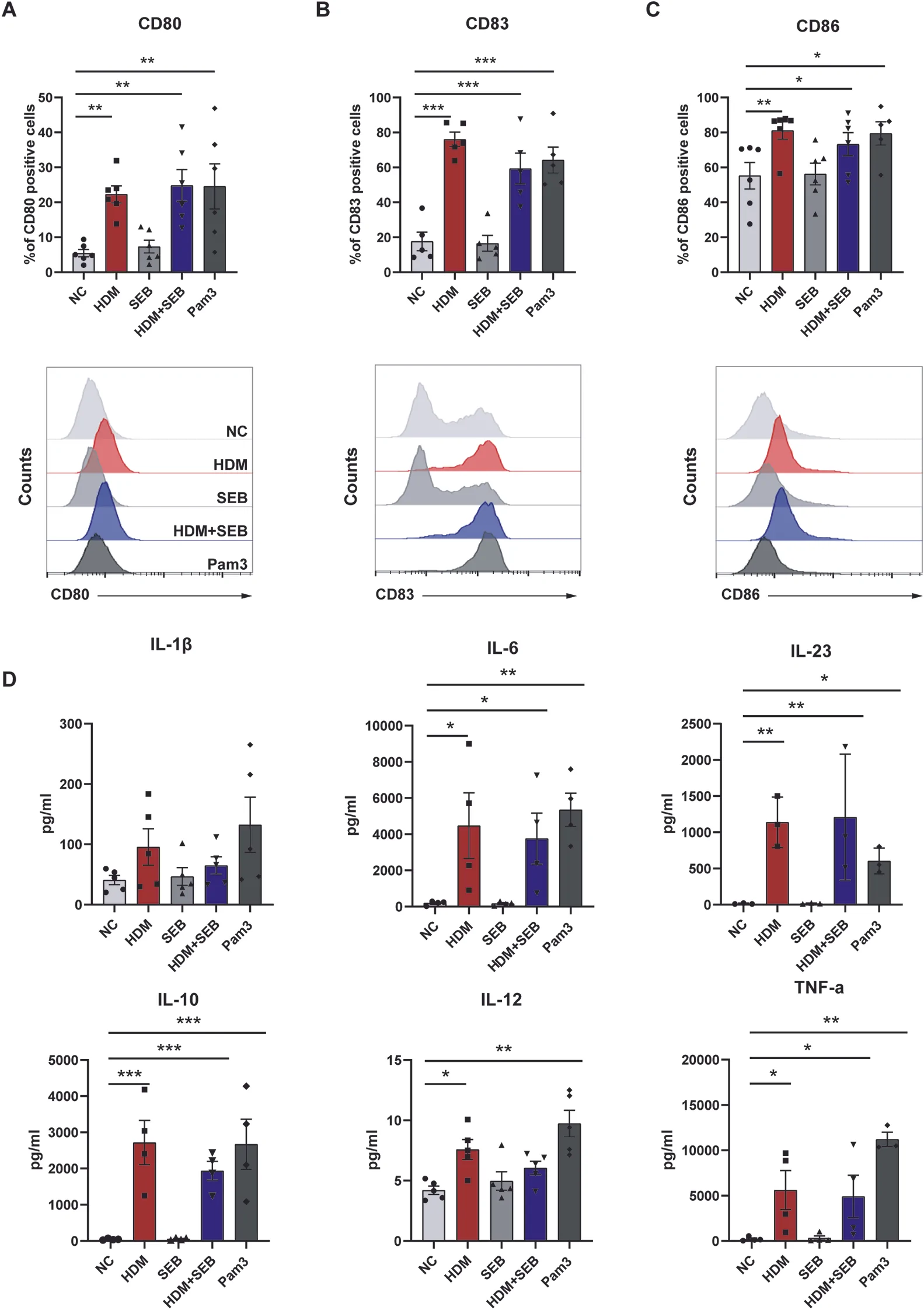
HDM up-regulated maturation markers and enhanced cytokine secretion. **(A–C)** Percentage of positive cells (CD80, CD83, CD86) after moIDECs stimulation with HDM alone or combined with SEB for 24 h. **(D)** HDM alone or combined with SEB stimulated moIDECs for 24 h. Collect the supernatant and use LEGENDplex™ to analyze the level of IL-1β, IL-6, IL-23, TNF-α, IL-10, IL- 12, IL-8 and MCP-1. No stimulation as negative control (NC). Pam3CSK4 was used as a positive control. Bar graphs show mean ± SEM for at least 3 experiments. The significance was calculated using the one-way ANOVA. *P < 0.05; **P < 0.01; ***P < 0.001.

### 3.5 HDM induced time-dependent maturation and function of moIDECs

Our initial findings established that a 24-hour HDM exposure potently activates moIDECs. However, the chronic nature of AD and the clinically observed phenomenon of antigen tolerance in a subset of patients prompted us to hypothesize that HDM’s influence on moIDECs might not be static but evolve over time. We profiled the functional and metabolic responses of moIDECs to HDM stimulation across an extended time course (6, 24, 48, and 72 h). Compared to the NC group, CD83 expression in the stimulated group exhibited a biphasic pattern, peaking at 48 h before declining, suggesting dynamic activation status of moIDECs upon HDM stimulation (**Figure 5A**). HDM stimulation significantly enhanced the production of both pro-inflammatory cytokines (IL-6, IFN-γ, IL-18, IL-12p70, TNF-α, IL-23) and the anti-inflammatory cytokine IL-10 in moIDECs compared to control groups (**Supplementary Figure S2**). Time-course analysis revealed that the ratios of pro- to anti-inflammatory cytokine (IL-6/IL-10, IFN-γ/IL-10, IL-18/IL-10, IL-12p70/IL-10, IL-23/IL-10 and TNF-α/IL-10) reached their peak levels at 6 h post-stimulation, followed by a gradual decline after 24 h (**Figure 5B**). These findings demonstrated that HDM-stimulated moIDECs initially adopt a pro-inflammatory phenotype, which is subsequently counterbalanced by enhanced IL-10-mediated anti-inflammatory regulation at later time points. At the same time, we also detected the mRNA expression of classical tolerogenic molecules (IDO1, PDL1, and ILT3). The results showed that under the stimulation of HDM, the expression of these molecules gradually increased with time (**Figure 5C**). This temporal regulation pattern suggests that the enhanced anti-inflammatory signals in moIDECs during the late phase help prevent excessive inflammatory responses.

**Figure 5:**
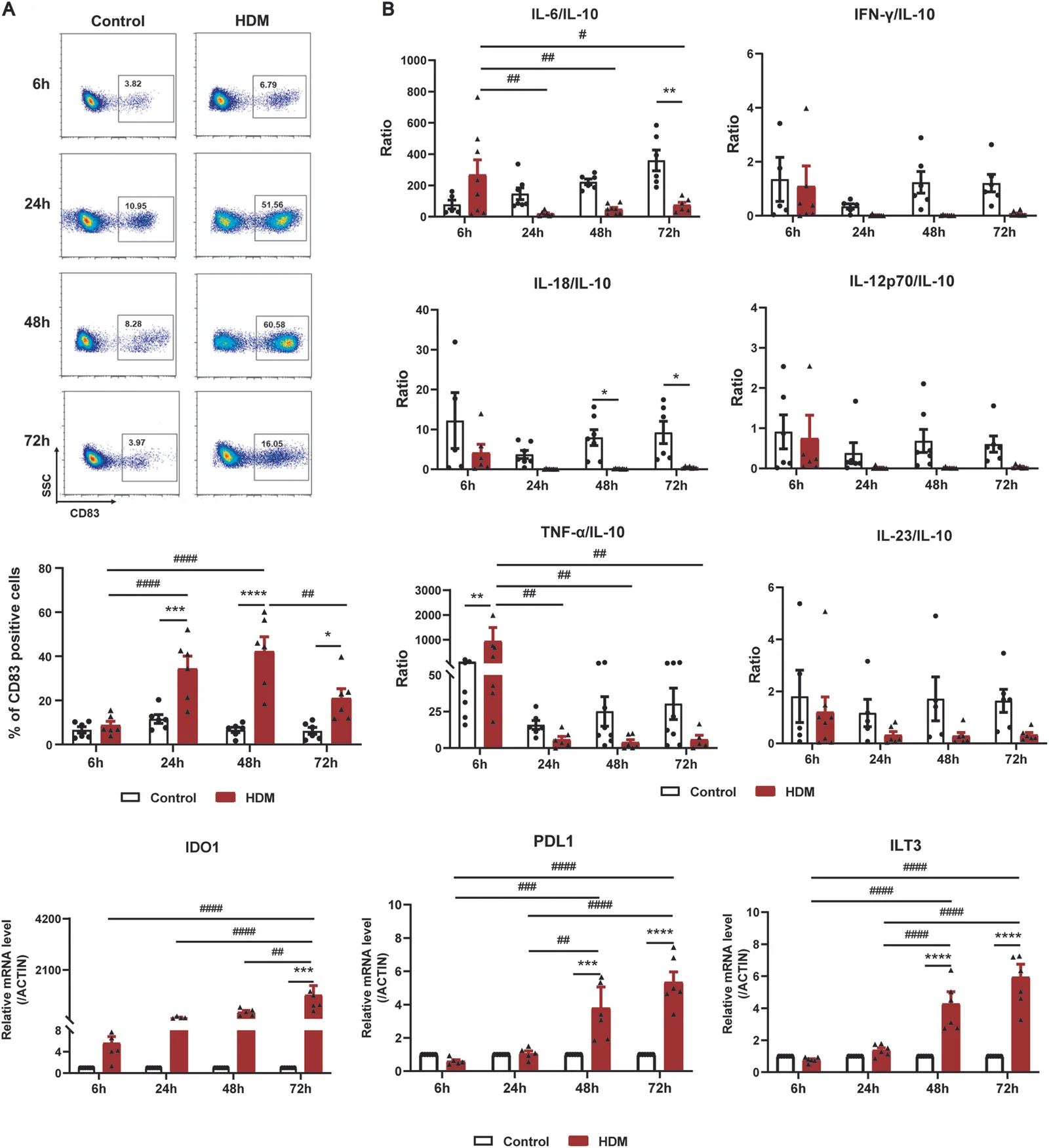
HDM induced time-dependent maturation and function. **(A)** Representative flow cytometry dot plots (upper panel) and quantitative analysis (lower panel) showing the percentage of CD83^+^ cells within moIDECs following HDM exposure for 6, 24, 48, and 72 h. **(B)** Time-course analysis of pro-inflammatory to anti-inflammatory cytokine ratios (IL-6/IL-10, IFN-γ/IL-10, IL-18/IL-10, IL-12p70/IL-10, TNF-α/IL-10, and IL-23/IL-10) in moIDECs stimulated with HDM versus control cells. **(C)** Relative mRNA expression levels (fold change) of classical tolerogenic molecules IDO1, PDL1, and ILT3 in moIDECs. White bars, control group; red bars, HDM-stimulated group. Bar graphs show mean ± SEM for at least 3 experiments. *p < 0.05, **p < 0.01, ***p < 0.001, and ****p < 0.0001 indicate significant differences between the HDM-stimulated group and the control group at the corresponding time point. ^#^p < 0.05, ^##^p < 0.01, ^###^p < 0.001, and ^####^p < 0.0001 indicate significant differences between consecutive time points within the HDM group.

To evaluate the immunomodulatory capacity of HDM-stimulated moIDECs at different time points, we performed allogeneic co-culture experiments with CD4^+^ T cells. HDM-treated moIDECs promoted biphasic CD4^+^ T cell proliferation, with significant enhancement at 24 h and 48 h (p< 0.05), followed by gradual attenuation (**Figure 6A**). Th1/2/17-associated cytokines (IFN-γ, IL-5, IL-13, IL-17) peaked at 24 h and subsequently declined. Treg-associated cytokines IL-10 showed sustained elevation, reaching maximal levels at 72 h (**Figure 6B**). These data suggest that HDM-activated moIDECs might initially potentiate Th1/Th2/Th17 cell proliferation, and then gradually shift toward an anti-inflammatory state associated with elevated IL-10 and tolerogenic molecules, which may support the generation of regulatory T cells (Tregs).

**Figure 6:**
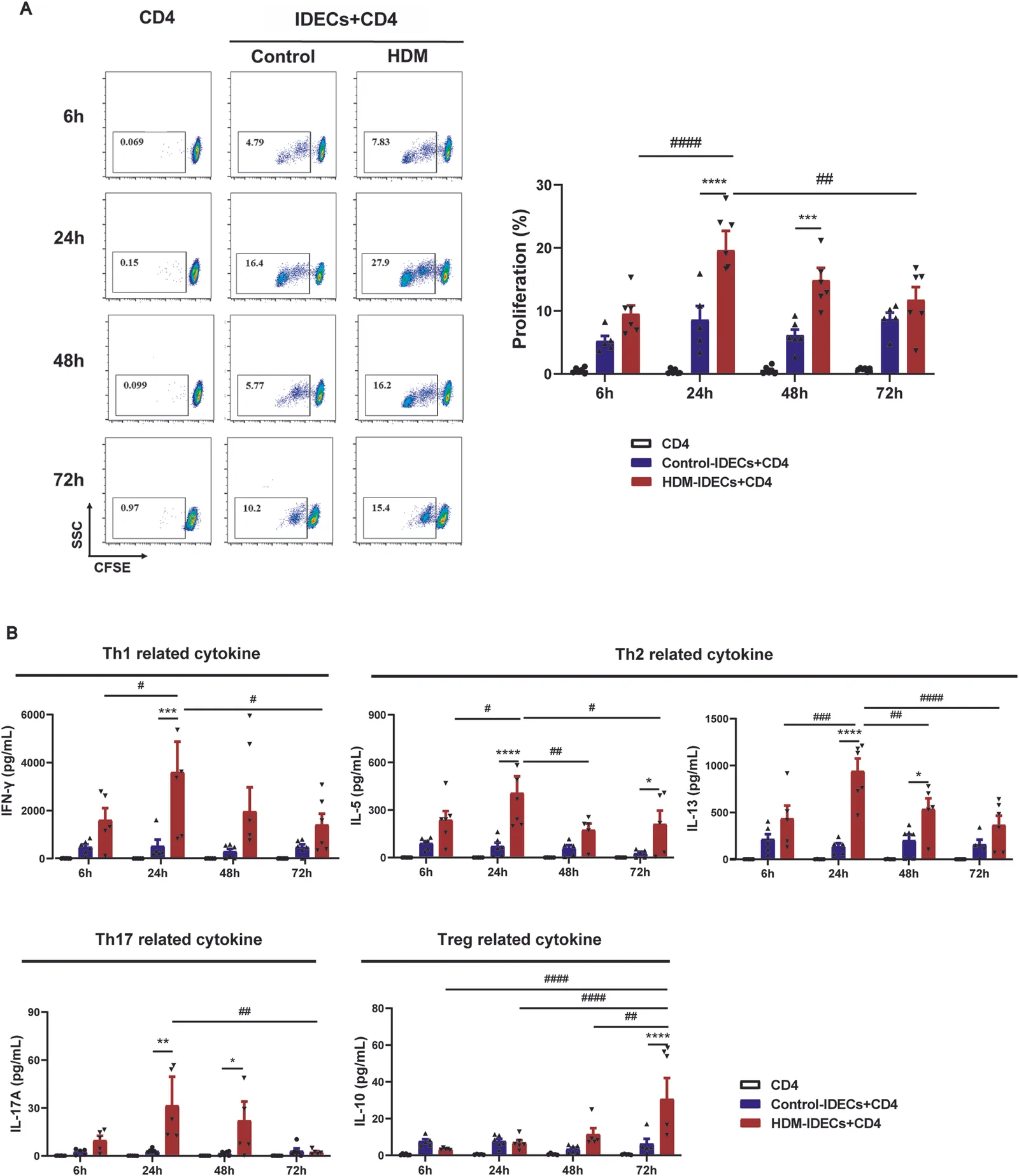
HDM-stimulated moIDECs time-dependently regulate CD4^+^ T cell proliferation and cytokine profiles in allogeneic co-cultures. **(A)** Representative CFSE dilution flow cytometry dot plots (left) and quantitative analysis (right) showing CD4^+^ T cell proliferation in allogeneic co-cultures with control or HDM-stimulated moIDECs at 6, 24, 48, and 72 h. **(B)** Concentrations of Th1-related (IFN-γ), Th2-related (IL-5 and IL-13), Th17-related (IL-17A), and Treg-related (IL-10) cytokines in the co-culture supernatants measured at the indicated time points. White bars. CD4^+^ T cells alone; blue bars, CD4^+^ T cells co-cultured with control moIDECs; red bars, CD4^+^ T cells co-cultured with HDM-stimulated moIDECs. Bar graphs show mean ± SEM for at least 3 experiments. *p < 0.05, **p < 0.01, ***p < 0.001, ****p < 0.0001 indicate significant differences between the HDM-moIDECs+CD4 group and the control moIDECs+CD4 group at the same time point. ^#^p < 0.05, ^##^p < 0.01, ^###^p < 0.001, ^####^p < 0.0001 indicate significant differences between consecutive time points within the HDM-moIDECs+CD4 group”.

### 3.6 Time-dependent metabolic adaption in HDM-stimulated moIDECs

To further elucidate whether the glucose metabolism exhibits dynamic changes in response to the maturation and function of moIDECs, we detected the expression of key enzyme related genes of the pathway. Compared to the negative control group, HDM stimulation significantly upregulated the mRNA expression of GLUT1and glycolytic rate-limiting enzymes-related genes (HK2, PFKL, and PKM) at 24 h (p < 0.01), but this upregulation plateaued thereafter, suggesting transient activation of glycolysis without further enhancement upon prolonged stimulation (**Figure 7A**). Interestingly, while gluconeogenic rate-limiting enzymes-related genes (FBP1, PCK2, and G6PC3) remained unchanged at early time points (6–24 h), their expression progressively increased during later stages (48–72 h) (**Figure 7B**). This metabolic shift implies that HDM-stimulated moIDECs may compensate energy demands through late-phase gluconeogenesis activation, potentially serving as a feedback mechanism to prevent cellular overactivation.

**Figure 7:**
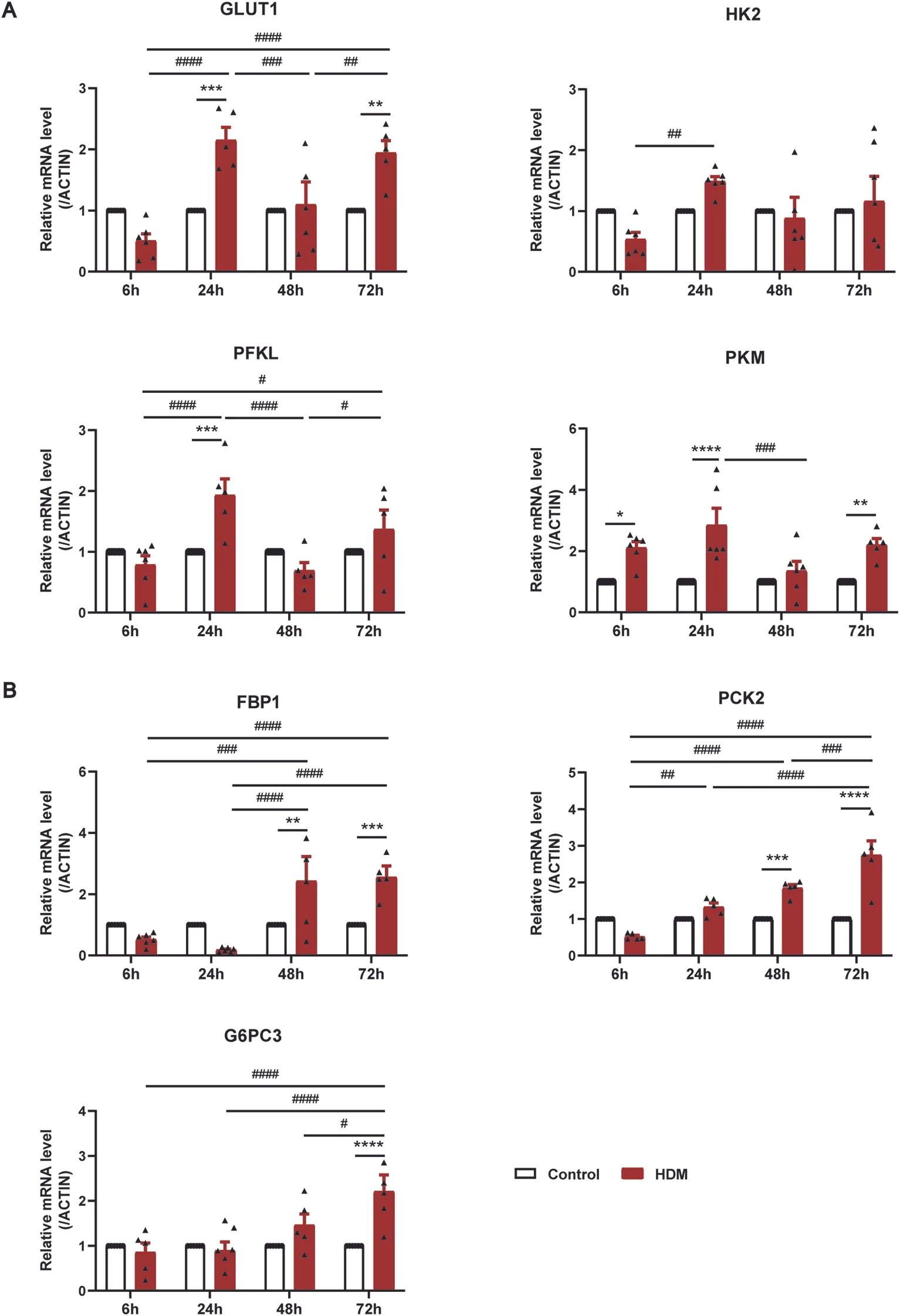
Time-dependent metabolic adaption in HDM-stimulated moIDECs. **(A)** Relative mRNA expression levels (fold change) of glycolysis-related genes (GLUT1, HK2, PFKL, and PKM), in moIDECs stimulated with HDM for 6, 24, 48, and 72 h. **(B)** Relative mRNA expression levels (fold change) of gluconeogenesis-related genes (FBP1, PCK2, and G6PC3) in moIDECs stimulated with HDM at the indicated time points. White bars, control group; red bars, HDM-stimulated group. Bar graphs show mean ± SEM for at least 3 experiments. *p < 0.05, **p < 0.01, ***p < 0.001, and ****p < 0.0001 indicate significant differences between the HDM-stimulated group and the control group at the corresponding time point. ^#^p < 0.05, ^##^p < 0.01, ^###^p < 0.001, and ^####^p < 0.0001 indicate significant differences between consecutive time points within the HDM group.

## 4 Discussion

Cellular metabolism is fundamental to immune cell function, and metabolism related protein levels correlate with AD severity (15), supported by transcriptomic and metabolomic evidence from AD patients (16–18). However, the specific role of cellular metabolism within the skin inflammatory environment—particularly in IDECs—remains unknown. To address this gap, this study aims to explore the metabolic characteristics of IDECs in AD and the immunometabolic regulation of moIDECs by two clinically relevant antigens: HDM and SEB. We found that inflammation- and metabolism-related genes of IDECs infiltrated in AD skin lesions are abnormally regulated, and multiple metabolic pathways are significantly dysregulated in IDECs. Among them, glycolysis was up-regulated and OXPHOS was down-regulated, suggesting that the skin inflammatory microenvironment can change the metabolic characteristics of IDECs. *In vitro* experiments demonstrated that, HDM stimulation of moIDECs led to enhanced glycolysis, which was accompanied by cell maturation and the secretion of inflammatory factors. In addition, HDM-induced immune response shows biphasic dynamics: early (6-24h) pro-inflammatory phase with IL-6, IFN-γ, IL-18, IL-12 TNF-α and IL-23 peaks, transitioning to late (48-72h) anti-inflammatory phase with elevated IL-10, classical tolerogenic molecules (IDO1, PDL1, and ILT3), a profile consistent with the potential to induce Tregs. and glycolysis peaks at 24h. In conclusion, our study provides further evidence for the hypothesis that glucose metabolism is involved in AD immune inflammation.

*In vitro*-generated moIDEC differ from moDC by higher surface FcϵRI expression and the presence of E-cadherin and CLA, which reflect an epidermal-resident phenotype. DC3 is a newly defined dendritic cell population that has been identified through high dimensional technologies. The typical phenotype of DC3 is CD1c^+^CD14^+^CD163^+^, among which CD163 is frequently used as a marker for the inflammatory DC3 subset. In the skin, DC3s are thought to correspond to CD14^+^ dendritic cells in the dermis, and they are recruited or expanded under inflammatory conditions. In atopic dermatitis, inflammatory monocyte derived dendritic cells (which may include DC3s) can be recruited to the skin. They may represent overlapping but not identical cell populations described under different experimental and conceptual frameworks.

AD is a chronic inflammatory skin disease driven by Th2-polarized immunity, in which dendritic cells, T cells, and keratinocytes synergistically amplify type 2 inflammation through IL-4, IL-5, and IL-13 release, disrupting the skin barrier and sustaining pruritus and infiltration3. While previous transcriptomic studies of AD skin biopsies have revealed broad abnormalities in inflammatory and barrier genes (19), the contributions of rare immune cell subsets such as IDECs are often obscured in bulk analyses. IDECs—identified through the marker genes CD1A, CD1C, FCER1A, and MRC1—were mainly contained within the DC-1 subpopulation and were significantly increased in the AD group. This selective expansion of the IDECs in AD lesions suggests that this population is specifically recruited in the inflammatory skin microenvironment. They highly expressed multiple pro-inflammatory genes (CCL17, MMP12, S100A8) in AD patients (20). Previous studies have shown that the above pro-inflammatory genes can be used as biomarkers that are strongly associated with the severity and clinical outcome of AD (21). Thus, we suggest that IDECs potentially contribute to the overall transcriptomic differences in AD. The high expression of CD86 means that IDECs in the AD group have a more mature and activated phenotype (22). Among all myeloid subsets, DC-1s showed the highest enrichment for Th2 differentiation, glycolysis, JAK–STAT, and HIF pathways, coupled with the lowest OXPHOS enrichment, indicating a glycolysis-biased metabolic reprogramming that may position DC-1s as key initiators of local type 2 responses. Conversely, Langerhans cells exhibited broad downregulation of most pathways, implying impaired antigen presentation and immune regulation, and a failure to restrain the overactive type 2 inflammation in AD lesions (**Supplementary Figure S3**).

We also found abnormal regulation of metabolism-related genes (LDHA, LIPA, GLUL) in the AD group’s IDECs, especially the LDHA encoding lactate dehydrogenase (LDH), one of the key enzymes of glycolytic (23). It is known that antigen-stimulated matured DCs switch to metabolic reprogramming characterized by aerobic glycolysis to meet dramatically increased energy demands (24). By comparing the metabolic profiles of IDECs in the two groups, we found that in the AD group, IDECs decreased activity of lipid metabolism and OXPHOS, as well as elevated activity of glycolysis. This suggests that IDECs appear to undergo metabolic reprogramming to adapt to the skin inflammatory microenvironment.

Dysregulation of IDECs metabolism in the AD group from scRNA-seq data was validated by *in vitro* moIDECs. HDM-stimulated moIDECs induced an increase of the glycolytic gene, the glycolytic product, and ECAR, as well as a decrease of the mitochondrial OCR. Guak et al. concluded that compared to LPS, BMDCs activated by the HDM exhibited a weaker pro-inflammatory phenotype (lower surface markers and lower inflammatory factors), with a rapid increase in glycolysis early (4 h) after stimulation, but lacked long-term (18 h) metabolic reprogramming (25). However, our study shows that HDM is not a weak agonist and its ability to induce moIDECs maturation is comparable to that of the positive control. Furthermore, HDM activation (24 h) of moIDECs also enhanced glycolysis significantly. These contradictory results can be explained by cellular model differences. The AD’s moIDECs express higher IgE high-affinity receptors (FcϵRI) than normal BMDCs (26), and activation of this receptor leads to rapid phosphorylation of tyrosine residues in M2-type pyruvate kinase (PKM2) thereby further promoting glycolysis (27, 28).

Previous research has demonstrated that in rheumatoid arthritis, the glycolytic serves as a critical driver of IL-1β production in dendritic cells, forming a pathogenic positive-feedback loop (29). Similarly, our data find that the early pro-inflammatory phase (6–24 h post-HDM stimulation) of moIDECs is marked by a robust upregulation of glycolytic flux and the concomitant secretion of cytokines such as TNF-α and IL-6. This result underscore glycolysis as a fundamental metabolic pathway supporting early inflammatory responses in AD skin lesions. Another pivotal finding of our study is the transition to a late phase (48–72 h) of moIDECs, where the metabolic profile shifts toward gluconeogenesis, coinciding with the upregulation of the anti-inflammatory cytokine IL-10, the expression of tolerogenic molecules (IDO1, PDL1, ILT3), and features suggestive of a potential to promote Treg. This temporal metabolic shift may underpin the dual role of moIDECs in AD via balancing immune activation (30). On one hand, it may explain why approximately half of AD patients eventually develop tolerance to HDM, as persistent antigen exposure drives moIDECs toward an anti- inflammatory phenotype. On the other hand, this anti- inflammatory state may not fully resolve inflammation but instead sustain a low-grade chronicity, predisposing patients to recurrent flares.

SEB is a superantigen whose primary mode of action requires direct bridging of the T cell receptor (TCR) and MHC class II molecules on antigen presenting cells, leading to potent T cell activation. The direct activation of DCs by SEB in the absence of T cells remains highly controversial. Therefore, the lack of direct moIDECs maturation or cytokine production upon SEB stimulation in our study (which used purified moIDECs without T cells) should not be overinterpreted as evidence. Mandron et al. suggested that SEB promotes IL-2 secretion and Th2 polarization by binding to TLR2 on the surface of moDCs (31). However, another study found that SEB alone did not activate BMDCs, but SEB promoted BMDCs maturation in the presence of ovalbumin (32). Consistent with the latter observation, SEB only upregulated IL-31 receptor expression in monocytes and macrophages, and did not affect cellular costimulatory molecules and inflammatory factors. However, SEB could promote CD86 expression as well as secretion of IL-1β, IL-6, and IL-18 by preemptively up-regulating the IL-31 receptor upon binding to IL-31 (33). These oppositions suggested that activation of myeloid cells by SEB might be indirect and dependent on preferential upregulation of other substances. As a superantigen, SEB primarily acted by bridging MHC class II and TCR Vβ regions, leading to polyclonal T-cell activation (5–20% of T cells) rather than directly stimulating APCs (34). In the absence of T cells, the lack of moIDECs maturation or cytokine production in our study was therefore expected. However, in the AD skin microenvironment where T cells are abundantly present, SEB may still modulate moIDECs function indirectly via T-cell-derived signals. Our results also suggest that although SEB-stimulated moIDECs do not affect mature molecules and inflammatory factors, SEB can reprogram glucose metabolism slightly. We hypothesize that this may be due to the cellular metabolic changes in SEB-stimulated moIDECs are not dramatic enough to induce the final mature phenotype.

In conclusion, our data establish HDM as a key driver of immunometabolic dysregulation in moIDECs, linking glucose metabolism to inflammatory responses in AD. These findings open avenues for targeted therapies modulating DC metabolism to restore immune homeostasis, but some limitations remain. Firstly, we acknowledge that the IDECs cluster may partially overlap with other moDC/DC3 subsets, which remains an unresolved challenge in the field, and future studies combining lineage tracing or multidimensional phenotypic analyses will be necessary to further refine the classification. Furthermore, the single-cell transcriptomic data show changes at the mRNA level. The expression of their key metabolic enzyme genes may not coincide with the proteins and metabolites. Secondly, *in vitro* culture conditions usually do not fully mimic the complexity of cells exposed to antigens *in situ*, nor do they correctly reflect the metabolic state *in vivo*. Finally, we speculated that metabolic dysregulation of cells might be involved in regulating the pathogenesis of AD, but it might also be a result of AD immune inflammation. Future studies using metabolic inhibitors or genetic approaches will be essential to establish causal relationships.

## Data Availability

The data provided in the study can be referenced at the https://ngdc.cncb.ac.cn/omix/. The data presented in the study are deposited in the Genome Sequence Archive for Human repository, accession number OMIX008321.
