# Supplementary material for: House dust mite drives biphasic immunomodulation of inflammatory dendritic epidermal cells via glucose metabolism in atopic dermatitis
Source: Front Immunol. 2026 Oct 12;17:1887390. doi: 10.3389/fimmu.2026.1887390 (PMC13696244; doi:10.3389/fimmu.2026.1887390)
Supplement: Supplementary file 1 [file SupplementaryFile1.docx]

Supplementary Material

# 1 Supplementary methods

1.1 **Collection and analysis of single-cell RNA transcriptome sequencing (scRNA-seq) data**

Seurat (4.0.1) was used for subsequent calculations of the downloaded count matrix in R (4.0.2). If the number of expressed genes was <200 or >6,000, the UMI count was <1,000, and/or the mitochondrial gene percentage was >0.1, cells were removed. The “IntegrateData” function was used to remove the batch effect across different samples. The “NormalizeData” and “ScaleData” functions were used to standardize and normalize the matrix for subsequent cell clustering and dimensionality reduction. The top 2,000 highly variable genes identified by the “FindVariableFeatures” function were used in the “RunPCA” function to perform principal component analysis. The “FindClusters” function was used to cluster the cells with a resolution of 1.5. The “RunUMAP” and “TsnePlot” functions were used to project the cells into a twodimensional space and visualize them. The “FindAllMarkers” function was used to identify differential expression genes (DEGs) compared with all other clusters. DEGs were defined as follows: detected in at least 25% of the cells, P<0.05 was based on the Wilcoxon rank sum test and Bonferroni correction, and | log10(fold change)| >0.3. Cell clusters were labeled through known classic markers. Cell subpopulations with high expression of marker genes CD1A, CD1C, MRC1, and FCER1A were identified as IDEC. The differentially expressed gene set of IDECs between the AD group and the HC group was obtained, and the Kyoto Encyclopedia of Genes and Genomes (KEGG) enrichment analysis was performed using the DAVID (v6.8) function. 8 metabolism-related pathways (glycolysis, OXPHOS, glucose energy metabolism, amino acid metabolism, nucleotide metabolism, pentose phosphate metabolism, lipid metabolism, glutamine metabolism) were collected from the KEGG database. Use the “AddModuleScore” function in Seurat to score the above metabolic pathways of IDEC, and use the Mann-Whitney rank sum test for statistical analysis. P < 0.05 is considered a statistically significant difference.

1.2 **Chemicals and reagents of metabolomics**

HPLC-grade acetonitrile (ACN) and methanol (MeOH) were purchased from Merck (Darmstadt, Germany). MilliQ water (Millipore, Bradford, USA) was used in all experiments. All of the standards were purchased from Sigma-Aldrich (St. Louis, MO, USA) and Zhenzhun etc. Formic acid was bought from Sigma-Aldrich (St. Louis, MO, USA). The stock solutions of standards were prepared at the concentration of 1 mg/mL in MeOH and other solutions. All stock solutions were stored at -20°C. The stock solutions were diluted with MeOH to working solutions before analysis.

1.3 **Sample preparation and extraction of metabolomics**

The sample was thawed on ice, 100 μL of ultrapure water extract was added to resuspend the cell pellet. Divide 50 μL cell suspension and add 200 μL of methanol (precooled at -20°C) and vortexed for 2 min under the condition of 2500 r/min. The sample was frozenin liquid nitrogen for 5 min, removed on ice for 5 min, after that, the sample was vortexed for 2 min.The previous step was repeated for 3 times. The sample was centrifuged at 12000 r/min for 10 min at 4°C. Take 200 μL of supernatant into a new centrifuge tube and place the supernatant in -20°C refrigerator for 30 min.Then the supernatant was centrifuged at 12000 r/min for 10 min at 4°C. After centrifugation, transfer 180 μL of supernatant through Protein Precipitation Plate for further LC-MS analysis. The left 50 μL cell suspension was frozen and thawed for 3 times, centrifuged at 12000 r/min for 10 min, and the supernatant was taken to determine the protein concentration by BCA Protein Assay kit.

1.4 **UPLC Conditions of metabolomics**

The sample extracts were analyzed using an LC-ESI-MS/MS system (Waters ACQUITY H-Class, https: //www.waters.com/nextgen/us/en.html; MS, QTRAP® 6500+ System, https://sciex.com /). The analytical conditions were as follows. Amide method: HPLC: column, ACQUITY UPLC BEH Amide (i.d.2.1×100 mm, 1.7 μm); solvent system, water with 10mM Ammonium acetate and 0.3% Ammonium hydroxide (A),90% acetonitrile/water(V/V)(B); The gradient was started at 95% B (0-1.2 min), decreased to 70% B (8 min),50% B (9-11 min), finaly ramped back to 95% B (11.1-15 min); flow rate, 0.4 mL/min; 40°C; injection volume: 2 μL.

1.5 **ESI-MS/MS Conditions of metabolomics**

AB 6500+ QTRAP® LC-MS/MS System, equipped with an ESI Turbo Ion-Spray interface, operating in both positive and negative ion modes and controlled by Analyst 1.6 software (AB Sciex). The ESI source operation parameters were as follows: ion source, turbo spray; source temperature 550°C; ion spray voltage (IS) 5500 V (Positive), -4500 V(Neagtive); curtain gas (CUR) were set at 35.0 psi; DP and CE for individual MRM transitions was done with further DP and CE optimization. A specific set of MRM transitions were monitored for each period according to the plant hormones eluted within this period.

1.6 **Differential metabolites selected**

Significantly regulated metabolites between groups were determined by VIP and absolute Log2FC (fold change). VIP values were extracted from OPLS-DA result, which also contain score plots and permutation plots, was generated using R package MetaboAnalystR. The data was log transform (log2) and mean centering before OPLS-DA. In order to avoid overfitting, a permutation test (200 permutations) was performed.

1.7 **KEGG annotation and enrichment analysis**

Identified metabolites were annotated using KEGG compound database (http://www.kegg.jp/kegg/ compound/), annotated metabolites were then mapped to KEGG Pathway database (http://www.kegg.jp/ kegg/pathway.html). Pathways with significantly regulated metabolites mapped to were then fed into MSEA (metabolite sets enrichment analysis), their significance was determined by hypergeometric test’s P-Values.

# 2 Supplementary Figure and Table

**2.1**

**Figure S1 2-DG blocks HDM-induced CD83 upregulation in moIDECs.**

moIDECs were treated with control medium (NC), NC plus 2-DG, HDM, or HDM plus 2-DG. CD83 expression was analyzed by flow cytometry. HDM significantly increased the percentage of CD83-positive cells (*** P < 0.001), and this effect was abolished by 2-DG (*** P < 0.001).

**Figure S2. HDM stimulation time-dependently enhanced pro-inflammatory and anti-inflammatory cytokine production in IDECs.**

IDECs were stimulated with HDM for 6, 24, 48, and 72 hours. The concentrations of pro-inflammatory cytokines (IL-6, IFN-γ, IL-18, TNF-α, IL-12p70, and IL-23) and the anti-inflammatory cytokine IL-10 in culture supernatants were determined. White bars, control group; red bars, HDM-stimulated group. Bar graphs show mean ± SEM for at least 3 experiments. * *p* < 0.05, ** *p* < 0.01, *** *p* < 0.001, and **** *p* < 0.0001 indicate significant differences between the HDM-stimulated group and the control group at the corresponding time point. # *p* < 0.05, ## *p* < 0.01, ### *p* < 0.001, and #### *p* < 0.0001 indicate significant differences between consecutive time point within the HDM group.

**Figure S3. Dot plot showing pathway enrichment scores of diverse functional biological pathways across distinct cell subpopulations from AD patients.**

Color gradient represents normalized pathway enrichment score: red indicates upregulated/enriched pathways (maximum score = 2), purple indicates downregulated/de-enriched pathways (minimum score = −2), and pale white denotes near-baseline enrichment (score = 0).

**2.2** qRT-PCR primers used in the current study.

| **Gene name** | **Primer sequence** |
| --- | --- |
| β-actin forward | CTCTTCCAGCCTTCCTTCCT |
| β-actin reverse | AGCACTGTGTTGGCGTACAG |
| GLUT1 forward | GGCCAAGAGTGTGCTAAAGAA |
| GLUT1 reverse | ACAGCGTTGATGCCAGACAG |
| HK2 forward | GAGCCACCACTCACCCTACT |
| HK2 reverse | CCAGGCATTCGGCAATGTG |
| PFKM forward | GAGTGACTTGTTGAGTGACCTCCAGAAA |
| PFKM reverse | CACAATGTTCAGGTAGCTGGACTTCG |
| FBP1 forward | TCAACTGCTTCATGCTGGAC |
| FBP1 reverse | CGTAGACCAGAGTGCGATGA |
| LDHA forward | TTGACCTACGTGGCTTGGAAG |
| LDHA reverse | GGTAACGGAATCGGGCTGAAT |
| MCT4 forward | CCATGCTCTACGGGACAGG |
| MCT4 reverse | GCTTGCTGAAGTAGCGGTT |
