# Supplementary material for: House dust mite drives biphasic immunomodulation of inflammatory dendritic epidermal cells via glucose metabolism in atopic dermatitis
Source: Front Immunol. 2026 Oct 12;17:1887390. doi: 10.3389/fimmu.2026.1887390 (PMC13696244; doi:10.3389/fimmu.2026.1887390)
Supplement: Supplementary file 2 [file Table1.docx]

The [International Society for the Advancement of Cytometry (ISAC)](https://isac-net.org/page/MIFlowCyt) has highlighted the importance of including comprehensive methodological information to ensure data reproducibility and reliability. In line with this, Frontiers in Immunology now requires authors to submit a checklist for manuscripts that involve flow or mass cytometry. This checklist helps standardize the reporting process, improving the quality and transparency of published data. By doing so, we support scientific progress, making it easier for other researchers to replicate and validate experiments.

This form should be submitted with any manuscripts using flow or mass cytometry.

Sample/specimen/material description

☐ Total blood

☐ PBMCs

☐ Organ digests

Other_ Inflammatory dendritic epidermal cells

Did the samples suffer any treatment before or after incubation with the antibodies?

Drug ____ House dust mites, staphylococcus aureus enterotoxin B ______________________________

☐ Cell permeabilization _____________________

☐ Dye ___________________________________

☐ Propidium iodine

☐ Not applicable

Other _____________________________________

Instrument and antibodies

Name of the Cytometer_ Gallios flow cytometer (Beckman, Miami, FL, USA)______________________________

| Antibodies and targets | Fluorochrome/ Metal | Catalog number/Company |
| --- | --- | --- |
| e.g. anti-CD4 | FITC | Cat. XXX/ XXX ltd. |
| anti-human CD83 | PE | 305308 Biolegend |

| anti-human CD86 | BrilliantViolet 421™ | 305426 Biolegend |
| --- | --- | --- |
| anti-human CD80 | FITC | 375405 Biolegend |
| anti-human CD1a | PE/Cyanine7 | 300122 Biolegend |
| anti-human CD14 | BrilliantViolet 510™ | 367123 Biolegend |
| anti-human CD206 | APC | 321110 Biolegend |
|  |  |  |
|  |  |  |
|  |  |  |
|  |  |  |
|  |  |  |
|  |  |  |
|  |  |  |
|  |  |  |
|  |  |  |
|  |  |  |
|  |  |  |
|  |  |  |
|  |  |  |
|  |  |  |
|  |  |  |
|  |  |  |
|  |  |  |
|  |  |  |
|  |  |  |
|  |  |  |
|  |  |  |
|  |  |  |

Data analyses

1. Name of the software___FlowJo (Treestar, Inc., Ashland, OR, USA)_____________________________
2. Reference gating strategy in the manuscript or supplementary material

Gating strategy in (eg Figure X)	Figure 3B, Figure S1A______________________
